# Scientific Items

**Published:** 1882-08-20

**Authors:** 


### SCIENTIFIC ITEMS.

**Brown Sugar Under the Microscope.**—Dr. Wm. Jones, in Medical Tribune, says: I will, with your permission, state what I have frequently seen in a few grains of raw, brown, unrefined sugar, when placed under an objective of four hundred diameters of the microscope.

I fear, however, the great objective power of the instrument will forever set you against all sweets, but especially that of sweetening your tea and coffee with brown sugar. The intensity of horror that came over me as I viewed the ugly-looking creatures, and observed the fearful battle that was going on in the few grains of sugar which lay before me, was anything but soothing to my nerves. It was such a sight as no one can conceive, who has not had an opportunity of observing what I will make an effort to describe. I have seen dozens, I am sure, of the most horrible-looking insects, in less than twenty grains of sugar, which appeared (under the power mentioned), as large as fleas, and somewhat resembling lobsters. Their legs or arms were provided with four joints each, with pinchers at the end, and covered with short, shining hair. Their heads were incased within a ring of ten or twelve spear-like *tentacula*, from the end of their nose (or what appeared to be their nose), had twelve to fifteen long, worm-like feelers, which they kept in continuous motion, for the purpose (it seemed to me), to ward off danger from any and all directions. And from their hinder-parts were presented ten or twelve spear-like bristles; I presume for protecting themselves from danger in that direction. Their bodies were about ⅛ of an inch in length, with dark lines on each side, similar to a head louse or *acarus scabies*, and they were in continual motion, throwing their ill-looking limbs in every direction, hitting their fetlows, who seemed to resent the freedom indulged in, and perfect bedlam seemed to exist among them, for I never saw them when they were not fighting one another, and tearing each other in a terrible manner. I do not know that these insects are natives of the sugar mentioned, or only invited from its want of cleanliness.

I have never been able to find any insects in refined sugar, but have observed them occasionally in the poorer quality of figs, and also in some prunes, and other foreign fruits.

**Transmission of Motive Power.**—We have already referred to the transmission of motive power from the mines to the centers where it is required, by means of electric wires instead of the transportation of coal as now practiced. Sir Henry Bessemer is credited with making a proposition of this kind with reference to London. He says:

"What a magnificent vista of legitimate mercantile enterprise this simple fact opens up to our country. Why should we not at once connect London with one of our nearest coal fields by means of a copper rod of one inch in diameter and capable of transmitting 84,000 horse-power to London, and practically bring up the coal by wire instead of by rail?"

Concerning this proposition an English exchange, assuming that each horse-power can be generated by consumption of 3 pounds of coal per hour, and that the engines work six days and a half per week, estimates "that there would be required an annual consumption of coal equal to 1,012,600 tons to produce the result. Now all this would, in the case assumed, be burned at the pit's mouth at a cost of, say, 6 shillings per ton for large and 2 shillings per ton for small coal; that is, at less than one-fourth the cost of coal in London. This would immensely reduce the cost of the electric light and of the motive power now used in London for such a vast variety of purposes. At the same time it would save the city from the enormous volumes of smoke and foul gas which this million of tons of coal would make if burned in the city."

Following up this train of thought the same authority argues that a 1-inch diameter copper rod would cost about £533 per mile, and if laid to a colliery 120 miles away, the interest at 5 per cent. on its first cost would be less than 1 penny per ton on the coal practically conveyed by it direct to the house consumer. These may appear to be visionary ideas at the present time, but with the rapid advance that is being made in electrical science, and in the appliances for utilizing the electric current, there is no saying what will be accomplished in the early future.—*Mechanical News.*

**Photo Printing in Colors.**—Some specimens of color printing in Herr Albert's studio deserve mention. Herr Albert has been experimenting with some success on the method which is usually connected with the name of Ducos du Hauron. A painting is photographed three times; the first negative is taken through a red screen, the second through a blue screen, and the third through a yellow screen. Albert employs colored liquids for his screens, and in this way he secures three negatives, in the taking of which respectively the rays of the three primary colors have been absorbed. The negative taken through the red screen is then printed upon red carbon tissue, and the other two negatives printed respectively with blue and yellow tissue. Then the three prints in red, yellow and blue are superposed, and the picture is finished. By working in this way Herr Albert claims to have reproduced a colored picture of Lemercier, which had been produced from eighteen stones (and therefore contained eighteen different tints), in all its prestine beauty. As the original was not at hand for comparison, it was impossible to say how far success had been secured, but certainly the Albert pictures are very pleasing and interesting.—*Exchange.*

**Dynamite.**—It is said that the 15 dynamite manufactories now under the control of M. Nobel turn out about 5,000 tons a year. In this country and in Europe it is estimated that the production of explosives containing nitro-glycerine is between 7,000 and 8,000 tons a year, and this quantity has the energy of at least 45,000 tons of ordinary gunpowder.—*Boston Jour. of Chem.*

